# Day 3 neutrophil-to-lymphocyte ratio and its derived indices predict 90-day poor outcomes following mechanical thrombectomy in acute ischemic stroke patients

**DOI:** 10.3389/fneur.2024.1496628

**Published:** 2024-12-18

**Authors:** Weiwei Gao, Arslan Annadurdyyev, Lingfeng Yu, Rong Huang, Bin Liu, Yixiong Lin, Huaiyi Li, Renjing Zhu

**Affiliations:** ^1^Department of Neurology, Zhongshan Hospital of Xiamen University, School of Medicine, Xiamen University, Xiamen, China; ^2^School of Medicine, Xiamen University, Xiamen, China; ^3^Department of Cardiology, National Regional Medical Center, Binhai Campus of the First Affiliated Hospital, Fujian Medical University, Fuzhou, China; ^4^Department of Radiology, The Second Affiliated Hospital of Xiamen Medical College, Xiamen, China

**Keywords:** acute ischemic stroke, mechanical thrombectomy, inflammatory markers, neutrophil-to-lymphocyte ratio, derived neutrophil-to-lymphocyte ratio, neutrophil-monocyte-to-lymphocyte ratio, prognosis

## Abstract

**Objective:**

To investigate the dynamic changes in neutrophil–to–lymphocyte ratio (NLR) and its derived indices following mechanical thrombectomy (MT) in patients with acute ischemic stroke (AIS) and evaluate their predictive value for prognosis.

**Methods:**

This single-center retrospective cohort study included AIS patients who underwent MT at Zhongshan Hospital of Xiamen University from January 2018 to February 2024. Peripheral blood samples were collected on admission, day 1, and day 3 after MT to determine the NLR, derived NLR (dNLR), and neutrophil–monocyte–to–lymphocyte ratio (NMLR). The primary endpoint was poor functional outcome at 90 days (modified Rankin scale score 3–6). The secondary endpoints included post-operative hemorrhagic transformation, malignant cerebral edema, in-hospital mortality, and 90-day all-cause mortality. Receiver operating characteristic (ROC) curve analysis was used to evaluate predictive performance, and multivariate logistic regression models were employed to explore the independent associations between inflammatory markers and prognosis.

**Results:**

A total of 423 eligible patients were included. Both groups showed similar dynamic trends in inflammatory markers, peaking on day 1 post-MT and subsequently declining. However, the poor outcome group (*n* = 255, 60.28%) maintained higher levels on day 3, whereas the good outcome group showed a significant decreasing trend. ROC curve analysis revealed that the NLR (AUC = 0.85, 95% CI: 0.81–0.89), dNLR (AUC = 0.86, 95% CI: 0.82–0.89), and NMLR (AUC = 0.85, 95% CI: 0.81–0.89) on day 3 post-MT had the strongest predictive power for 90-day poor outcomes. After comprehensive adjustment for confounders, these inflammatory markers were independently associated with 90-day poor outcomes: for each unit increase in the NLR, the risk of poor outcome increased by 38% (OR = 1.38, 95% CI: 1.28–1.49, *p* < 0.001); for dNLR, it increased by 104% (OR = 2.04, 95% CI: 1.73–2.40, *p* < 0.001); and for NMLR, it increased by 35% (OR = 1.35, 95% CI: 1.26–1.45, *p* < 0.001).

**Conclusion:**

Inflammatory markers (NLR, dNLR, and NMLR) on day 3 post-MT can serve as independent predictors of prognosis in AIS patients treated with MT. Dynamic monitoring of inflammatory markers may facilitate early risk stratification and guide individualized treatment strategies.

## 1 Introduction

Acute ischemic stroke (AIS) is a leading cause of mortality and disability worldwide. According to statistics from the World Health Organization, approximately 12 million new stroke cases occur globally each year, with ischemic strokes accounting for a staggering 62.4%, resulting in nearly 6 million deaths or severe disabilities (1). Large vessel occlusion (LVO) strokes constitute one-third of AIS cases and often present with significant neurological impairments and poorer prognoses (2).

In recent years, mechanical thrombectomy (MT) has emerged as the standard treatment for LVO patients because of its remarkable clinical benefits (3). Compared with intravenous thrombolysis alone, MT significantly improves functional outcomes and reduces mortality and disability rate (4). However, despite advancements in MT techniques, a considerable proportion of patients still experience unfavorable outcomes. A prospective study revealed that among LVO patients undergoing MT, only 49% reached functional independence (defined as a modified Rankin Scale score [mRS] ≤ 2) at 90 days (5). Poor outcomes not only increase the burden on patients and their families but also place substantial pressure on society and healthcare systems. Therefore, identifying key factors influencing MT outcomes is crucial for early recognition of high-risk patients and optimization of clinical management strategies.

Inflammatory responses play a pivotal role in the occurrence, progression, and prognosis of AIS (6). Ischemic brain injury rapidly activates the innate immune responses, leading to the release and recruitment of inflammatory cells and mediators, which further exacerbates brain tissue damage and disrupts blood–brain barrier (BBB) integrity (7). The neutrophil-to-lymphocyte ratio (NLR), an emerging inflammatory marker in peripheral blood, has been shown to be closely associated with the prognosis of AIS patients (8, 9). However, previous studies have primarily focused on baseline levels or single measurements of the NLR, and a systematic evaluation of its dynamic changes and prognostic value is lacking.

Furthermore, the derived neutrophil-to-lymphocyte ratio (dNLR) and neutrophil-monocyte-to-lymphocyte ratio (NMLR), which are derived indices of the NLR, have demonstrated significant prognostic predictive value in various inflammatory and immune diseases (10, 11) as well as acute myocardial infarction (12). Nevertheless, the clinical utility of these novel inflammatory markers in AIS patients has not been fully validated, and whether their predictive performance is superior to that of the traditional NLR remains to be elucidated.

Given this background, we conducted this retrospective cohort study to investigate the dynamic changes in NLR and its derived indices following MT in AIS patients and evaluate their prognostic predictive value. By systematically assessing the dynamic changes in peripheral immune markers, this study aimed to provide new clinical insights into prognostic assessment for AIS patients and evidence-based guidance for individualized treatment strategies.

## 2 Materials and methods

### 2.1 Study design and population

This single-center retrospective cohort study consecutively enrolled AIS patients who underwent MT at Zhongshan Hospital of Xiamen University from January 2018 to February 2024. The inclusion criteria were: (1) age ≥ 18 years; (2) met the clinical and imaging diagnostic criteria for AIS established by the World Health Organization, and (3) CT angiography at admission confirmed the presence of intracranial large vessel occlusion, including internal carotid artery, middle cerebral artery (M1 or M2 segment), basilar artery, or posterior cerebral artery (P1 segment). The exclusion criteria were as follows: (1) baseline CT or magnetic resonance imaging indicating intracranial hemorrhage; (2) pre-stroke mRS score ≥ 2; (3) severe systemic diseases such as renal failure, severe liver dysfunction, or malignant tumors; (4) presence of infectious diseases, inflammatory diseases, immune system disorders, or ongoing immunotherapy on admission; (5) comorbidities that may affect inflammatory markers, including tumors, myocardial infarction, trauma, recent surgery, or allergic reactions; (6) lack of complete laboratory data, and (7) lack of follow-up data. During the screening process, 78 patients who did not meet the criteria were excluded, 14 of whom were not included because of incomplete data related to death within 3 days. A total of 423 patients were ultimately included in the analysis.

### 2.2 Data collection

A standardized electronic data collection form was used to gather patients’ clinical data, including demographic characteristics (age, sex), medical history (hypertension, diabetes, hyperlipidemia, atrial fibrillation, valvular heart disease, coronary artery disease, smoking history, and alcohol consumption history), admission assessments (National Institutes of Health Stroke Scale [NIHSS] score, Glasgow Coma Scale [GCS] score, and blood pressure), vessel occlusion site, stroke etiology, intravenous thrombolysis status, time metrics (onset-to-puncture time, onset-to-recanalization time, and puncture-to-recanalization time), and MT procedural parameters (number of thrombectomy attempts, retrieval technique, and vessel recanalization status).

Venous blood samples were collected on admission, and on days 1 and 3 post-MT. White blood cell counts and differentials were determined using an automated hematology analyzer. The inflammatory marker calculation formulas were as follows:

NLR = neutrophil count/lymphocyte count.

dNLR = neutrophil count/(white blood cell count – neutrophil count).

NMLR = (monocyte count + neutrophil count)/lymphocyte count.

All relevant data were independently collected and recorded by two trained neurologists following a standardized protocol and cross-checked by other researchers to ensure data accuracy and completeness.

### 2.3 Definitions

The primary endpoint was poor outcome at 90 days, defined as a mRS score of 3–6. The secondary outcomes included postoperative hemorrhagic transformation (HT), malignant cerebral edema, in-hospital mortality, and 90-day all-cause mortality. Vessel recanalization status was assessed on the basis of immediate post-MT cerebral angiography results using the modified Thrombolysis in Cerebral Infarction (mTICI) scale, with mTICI grades 2b-3 defined as successful recanalization (13). HT was identified as new intracranial hemorrhage detected on postoperative imaging (CT/MRI). Patients routinely underwent follow-up head CT at 24 h post-MT, and additional CT/MRI was performed within 72 h post-MT if neurological deterioration occurred (14). Malignant cerebral edema was defined as significant space-occupying effect (midline shift ≥ 5 mm) in the infarct area on imaging within 72 h post-MT.

All patients underwent follow-up assessments 90 days after stroke onset, which were conducted by specially trained research coordinators through telephone interviews. During the follow-up, we collected information on patients’ functional status and all-cause mortality. For deceased patients, we first obtained preliminary information from the patients’ relatives and then further verified it through death certificates from the primary hospital or the patients’ community hospital to ensure data accuracy and completeness. To ensure the consistency and reliability of the assessments, all follow-up evaluations were performed by rigorously trained personnel. The assessment results were entered in real-time into the National Cerebrovascular Disease Big Data Platform (Stroke Center Construction Information Management System) for centralized data management and analysis.

### 2.4 Statistical analysis

Statistical analyses were performed using R software (version 4.2.2). The Shapiro–Wilk test was used to assess the normality of continuous variables. Normally distributed continuous variables are expressed as the mean ± standard deviation (mean ± SD), and comparisons between groups were performed using the independent samples *t*-test. Non-normally distributed continuous variables were expressed as median and interquartile range [median (Q1, Q3)], and the Mann–Whitney *U* test was used for inter-group comparisons. Categorical variables were expressed as frequencies and percentages [*n* (%)], and analyzed using Pearson’s chi-square test or Fisher’s exact test.

To evaluate the predictive performance of inflammatory markers, we analyzed the predictive value of the NLR, dNLR, and NMLR at three time points (at admission, 24 h, and 72 h post-MT) for the primary and secondary endpoints. Receiver operating characteristic (ROC) curves were constructed, and the area under the curve (AUC) and its 95% confidence interval were calculated. The optimal cutoff value was determined via Youden’s index (maximum value of sensitivity + specificity – 1), and the corresponding sensitivity, specificity, positive predictive value, and negative predictive value were calculated.

To explore the independent associations between inflammatory markers and 90-day functional outcomes, we constructed three stepwise adjusted logistic regression models: Model A was unadjusted; Model B was adjusted for age, hyperlipidemia, and atrial fibrillation; Model C was further adjusted for the baseline NIHSS score, GCS score, and number of mechanical thrombectomy attempts based on Model B; and the covariates included in the models were based on the results of univariate analysis (*p* < 0.05). All the statistical tests were two-sided, and *p* < 0.05 was considered statistically significant.

## 3 Results

### 3.1 Baseline characteristics

This study ultimately included 423 AIS patients who underwent MT (Table 1), with a median age of 68.00 years; 66.43% were male. The median NIHSS score on admission was 15, and the median GCS score was 12. Hypertension (68.79%) was the most common risk factor, followed by atrial fibrillation (40.90%), smoking history (34.99%), diabetes (29.79%), and hyperlipidemia (23.88%). Large artery atherosclerosis (52.01%) and cardioembolism (43.26%) were the main stroke etiologies. Most patients had anterior circulation occlusion (84.16%), and 40.90% received intravenous thrombolysis. The median onset-to-puncture time was 378 min, the puncture-to-recanalization time was 98 min, and the onset-to-recanalization time was 499 min. The median number of thrombectomy attempts was 2.00 (IQR, 1.00–2.00), and 38.53% of patients achieved successful recanalization (mTICI score 2b-3).

**Table 1:** Baseline characteristics and clinical features of patients between good and poor outcome groups.

Variables	Total (*n* = 423)	Good outcome (*n* = 168)	Poor outcome (*n* = 255)	*p*-value
Age, year	68.00 (57.00, 76.00)	64.00 (55.00, 71.25)	70.00 (59.00, 78.00)	**<0.001**
Sex, male, *n* (%)	281 (66.43)	116 (69.05)	165 (64.71)	0.355
Current smoker, *n* (%)	148 (34.99)	68 (40.48)	80 (31.37)	0.055
Alcohol consumption, *n* (%)	93 (21.99)	41 (24.40)	52 (20.39)	0.330
Baseline NIHSS score	15.00 (11.00, 19.00)	13.00 (8.00, 16.00)	17.00 (13.00, 20.00)	**<0.001**
Baseline GCS score	12.00 (9.00, 14.00)	14.00 (11.00, 15.00)	11.00 (8.00, 14.00)	**<0.001**
Systolic blood pressure	148.00 (133.00, 164.00)	148.00 (133.00, 162.00)	149.00 (132.50, 166.00)	0.641
Diastolic blood pressure	87.00 (77.00, 97.00)	87.00 (76.75, 97.25)	88.00 (77.00, 96.00)	0.917
Medical history, *n* (%)				
Diabetes mellitus	126 (29.79)	42 (25.00)	84 (32.94)	0.081
Hypertension	291 (68.79)	110 (65.48)	181 (70.98)	0.232
Dyslipidemia	101 (23.88)	50 (29.76)	51 (20.00)	**0.021**
Prior stroke or TIA	66 (15.60)	24 (14.29)	42 (16.47)	0.545
Atrial Fibrillation	173 (40.90)	57 (33.93)	116 (45.49)	**0.018**
Coronary artery disease	52 (12.29)	16 (9.52)	36 (14.12)	0.159
Valvular heart disease	53 (12.53)	19 (11.31)	34 (13.33)	0.538
Stroke etiology, *n* (%)				0.209
Large-artery atherosclerosis	220 (52.01)	96 (57.14)	124 (48.63)	
Cardioembolism	183 (43.26)	64 (38.10)	119 (46.67)	
Other determined etiology	20 (4.73)	8 (4.76)	12 (4.71)	
Occluded vessel, *n* (%)				
Anterior circulation	356 (84.16)	142 (84.52)	214 (83.92)	0.868
Posterior circulation	72 (17.02)	25 (14.88)	47 (18.43)	0.342
Treatment characteristics				
Intravenous thrombolysis, *n* (%)	173 (40.90)	72 (42.86)	101 (39.61)	0.506
Onset-to-puncture time, min	378.00 (263.50, 595.00)	368.50 (233.75, 661.25)	385.00 (275.00, 579.00)	0.457
Puncture-to-recanalization time, min	98.00 (61.00, 155.50)	94.50 (54.75, 141.50)	100.00 (67.50, 165.00)	0.060
Onset-to-recanalization time, min	499.00 (363.00, 737.50)	494.00 (319.50, 755.25)	500.00 (377.50, 723.50)	0.422
Number of thrombectomy attempts	2.00 (1.00, 2.00)	1.00 (1.00, 2.00)	2.00 (1.00, 3.00)	**0.001**
mTICI score 2b-3, *n* (%)	163 (38.53)	73 (43.45)	90 (35.29)	0.092
Thrombectomy technique, *n* (%)				
Stent retriever	105 (24.82)	43 (25.60)	62 (24.31)	0.765
Aspiration	24 (5.67)	9 (5.36)	15 (5.88)	0.819
Combined	260 (61.47)	104 (61.90)	156 (61.18)	0.880
Clinical outcomes and complications, *n* (%)				
Discharge mRS score	4.00 (2.00, 5.00)	2.00 (1.00, 3.00)	5.00 (4.00, 5.00)	**<0.001**
90-day mRS score	3.00 (1.00, 5.00)	1.00 (0.00, 2.00)	4.00 (4.00, 6.00)	**<0.001**
Hemorrhage transformation	197 (46.57)	51 (30.36)	146 (57.25)	**<0.001**
Malignant cerebral edema	55 (13.00)	0 (0.00)	55 (21.57)	**<0.001**
In-hospital mortality	43 (10.17)	0 (0.00)	43 (16.86)	**<0.001**
90-day mortality	86 (20.33)	0 (0.00)	86 (33.73)	**<0.001**

Bold *p*-values indicate statistical significance (*P* < 0.05). NIHSS, National Institutes of Health Stroke Scale; GCS, Glasgow Coma Scale; TIA, Transient Ischemic Attack; mTICI, modified Thrombolysis in Cerebral Infarction; mRS, modified Rankin Scale.

The 90-day follow-up results revealed that 168 (39.72%) patients had good functional outcomes (mRS 0–2), whereas 255 (60.28%) patients had poor functional outcomes (mRS 3–6). Compared with those in the good outcome group, patients in the poor outcome group were older, had higher baseline NIHSS scores, lower baseline GCS scores (all *p* < 0.001), and required more retrieval attempts (*p* = 0.001). Patients in the poor outcome group had a lower proportion of hyperlipidemia (*p* = 0.021) but a higher proportion of atrial fibrillation (*p* = 0.018). There were no statistically significant differences in the other characteristics between the two groups (all *p* > 0.05).

The median 90-day mRS score for the entire cohort was 3.00, with 1.00 in the good outcome group and 4.00 in the poor outcome group (*p* < 0.001). The incidence of hemorrhagic transformation was significantly higher in the poor outcome group (57.25% vs. 30.36%, *p* < 0.001). Fifty-five (13.00%) patients developed malignant cerebral edema, and 43 (10.17%) patients died during hospitalization. Eighty-six (20.33%) patients died within 90 days.

### 3.2 Dynamic changes in the NLR and its derived indices

**Figure 1:**
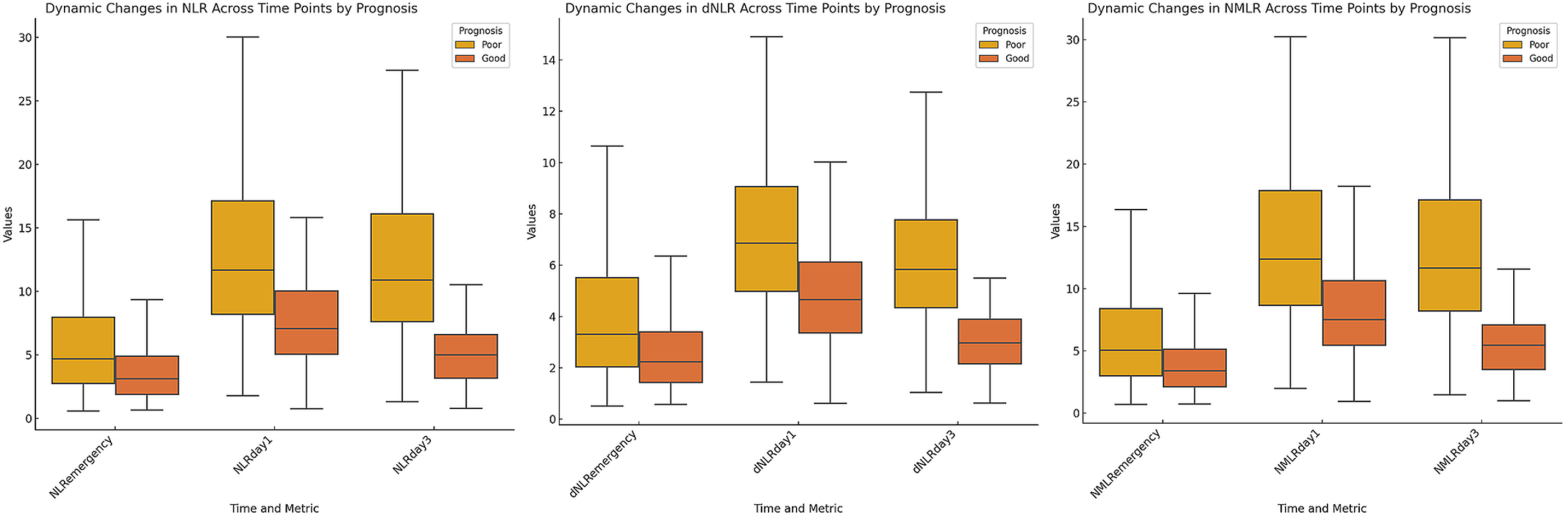
Dynamic changes in inflammatory markers stratified by 90-day poor outcome. Box plots depicting the temporal profiles of neutrophil-to-lymphocyte ratio, derived neutrophil-to-lymphocyte ratio, and neutrophil-monocyte-to-lymphocyte ratio at three time points: on admission, day 1 post-MT, and day 3 post-MT. Patients were stratified by 90-day functional outcome.

We evaluated the dynamic changes in the NLR, dNLR, and NMLR at different time points (Figure 1). The results revealed that the levels of inflammatory markers were consistently higher in the poor outcome group than in the good outcome group (all *p* < 0.001) (Table 2). Further analysis revealed that the median NLR, dNLR, and NMLR in the poor outcome group peaked on day 1 post-MT [NLR: 11.66 (8.18, 17.12) vs. 7.05 (5.05, 10.01); dNLR: 6.86 (4.99, 9.06) vs. 4.66 (3.36, 6.12); NMLR: 12.38 (8.66, 17.88) vs. 7.53 (5.46, 10.63), all *p* < 0.001]. Although they decreased on day 3 post-MT, the poor outcome group still maintained higher levels [NLR: 10.89 (7.61, 16.08) vs. 5.00 (3.16, 6.58); dNLR: 5.84 (4.35, 7.77) vs. 2.98 (2.16, 3.90); NMLR: 11.64 (8.19, 17.13) vs. 5.46 (3.52, 7.10), all p < 0.001].

**Table 2:** Comparison of laboratory parameters at different time points between good and poor outcome groups.

Variables	Total (*n* = 423)	Good outcome (*n* = 168)	Poor outcome (*n* = 255)	*P-*value
WBC day 0	8.53 (6.74, 10.98)	8.18 (6.65, 10.09)	8.80 (6.75, 11.94)	**0.028**
Neutrophils day 0	6.09 (4.36, 8.57)	5.45 (4.01, 7.42)	6.44 (4.58, 9.74)	**<0.001**
Lymphocytes day 0	1.49 (1.04, 2.17)	1.75 (1.21, 2.42)	1.37 (0.94, 1.97)	**<0.001**
Monocytes day 0	0.45 (0.35, 0.58)	0.43 (0.35, 0.58)	0.46 (0.35, 0.56)	0.898
NLR day 0	4.08 (2.34, 6.75)	3.11 (1.87, 4.90)	4.68 (2.74, 7.95)	**<0.001**
dNLR day 0	2.85 (1.80, 4.70)	2.24 (1.43, 3.40)	3.31 (2.04, 5.51)	**<0.001**
NMLR day 0	4.37 (2.60, 7.07)	3.40 (2.11, 5.14)	5.06 (3.01, 8.40)	**<0.001**
WBC day 1	11.71 (9.80, 14.01)	10.58 (8.66, 12.57)	12.49 (10.34, 14.69)	**<0.001**
Neutrophils day 1	9.99 (8.02, 12.03)	8.55 (6.91, 10.57)	10.73 (8.83, 12.93)	**<0.001**
Lymphocytes day 1	1.05 (0.74, 1.38)	1.23 (0.92, 1.62)	0.93 (0.70, 1.21)	**<0.001**
Monocytes day 1	0.60 (0.46, 0.74)	0.58 (0.46, 0.71)	0.61 (0.46, 0.79)	0.113
NLR day 1	9.68 (6.45, 13.90)	7.05 (5.05, 10.01)	11.66 (8.18, 17.12)	**<0.001**
dNLR day 1	5.86 (4.23, 7.84)	4.66 (3.36, 6.12)	6.86 (4.99, 9.06)	**<0.001**
NMLRay 1	10.29 (6.90, 14.79)	7.53 (5.46, 10.63)	12.38 (8.66, 17.88)	**<0.001**
WBC day 3	10.62 (8.39, 13.66)	8.75 (7.05, 10.66)	12.13 (9.66, 15.60)	**<0.001**
Neutrophils day 3	8.60 (6.39, 11.41)	6.38 (4.97, 8.24)	10.07 (8.11, 13.85)	**<0.001**
Lymphocytes day 3	1.16 (0.86, 1.48)	1.37 (1.15, 1.69)	1.02 (0.73, 1.27)	**<0.001**
Monocytes day 3	0.68 (0.49, 0.85)	0.61 (0.45, 0.77)	0.72 (0.54, 0.91)	**<0.001**
NLR day 3	7.82 (4.80, 12.97)	5.00 (3.16, 6.58)	10.89 (7.61, 16.08)	**<0.001**
dNLR day 3	4.50 (2.95, 6.65)	2.98 (2.16, 3.90)	5.84 (4.35, 7.77)	**<0.001**
NMLR day 3	8.38 (5.19, 13.75)	5.46 (3.52, 7.10)	11.64 (8.19, 17.13)	**<0.001**

Bold *P*-values indicate statistical significance (*P* < 0.05). WBC, white blood cell count; NLR, neutrophil-to-lymphocyte ratio; dNLR, derived neutrophil-to-lymphocyte ratio; NMLR, neutrophil-monocyte-to-lymphocyte ratio. Reference ranges: WBC 3.5–9.5 × 10^9/L, Neutrophils 1.8–6.3 × 10^9/L, Lymphocytes 1.1–3.2 × 10^9/L, Monocytes 0.10–0.60 × 10^9/L.

### 3.3 Predictive performance analysis

**Figure 2:**
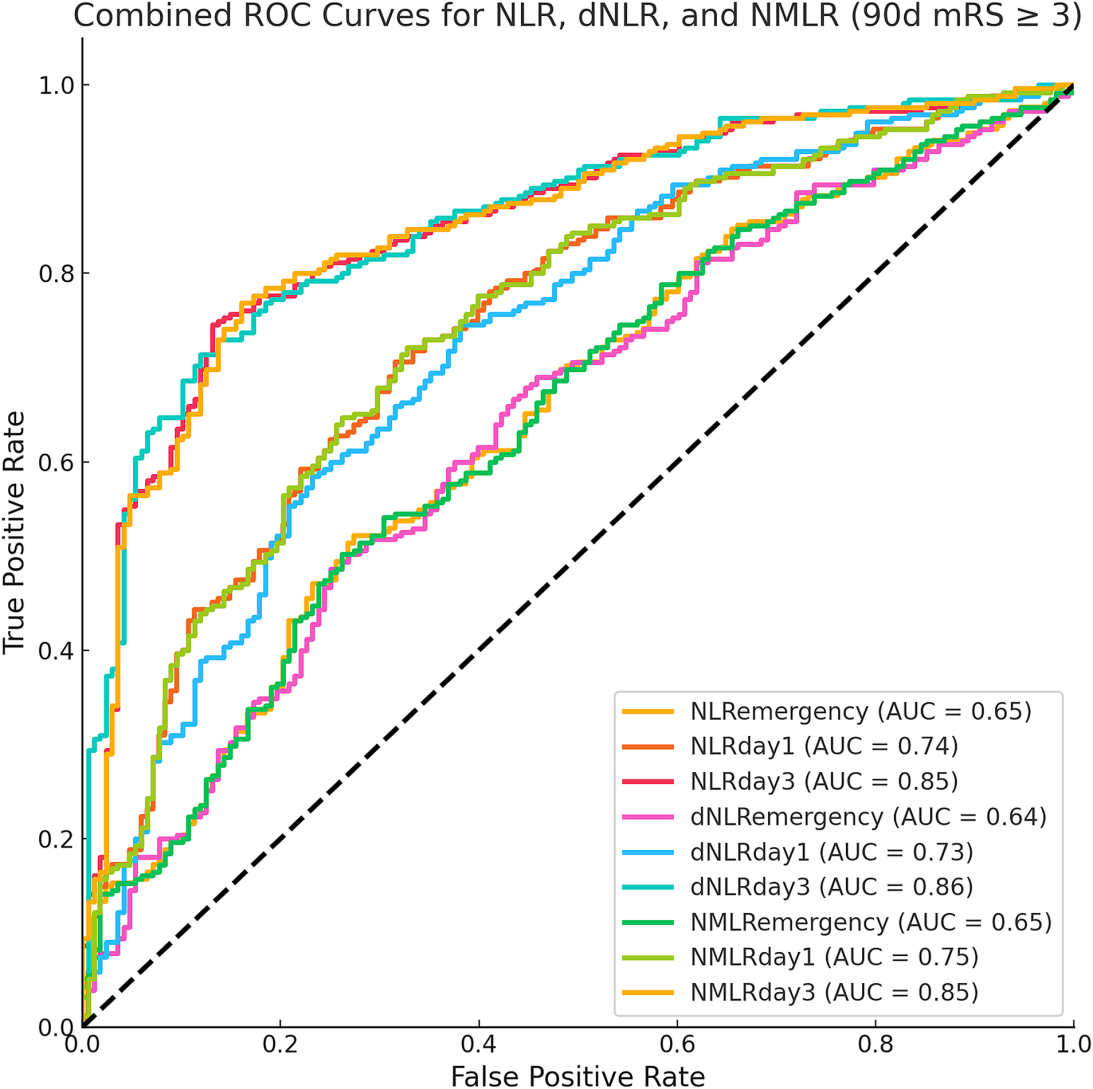
Receiver operating characteristic curves for inflammatory markers in predicting 90-day poor functional outcome.

**Figure 3:**
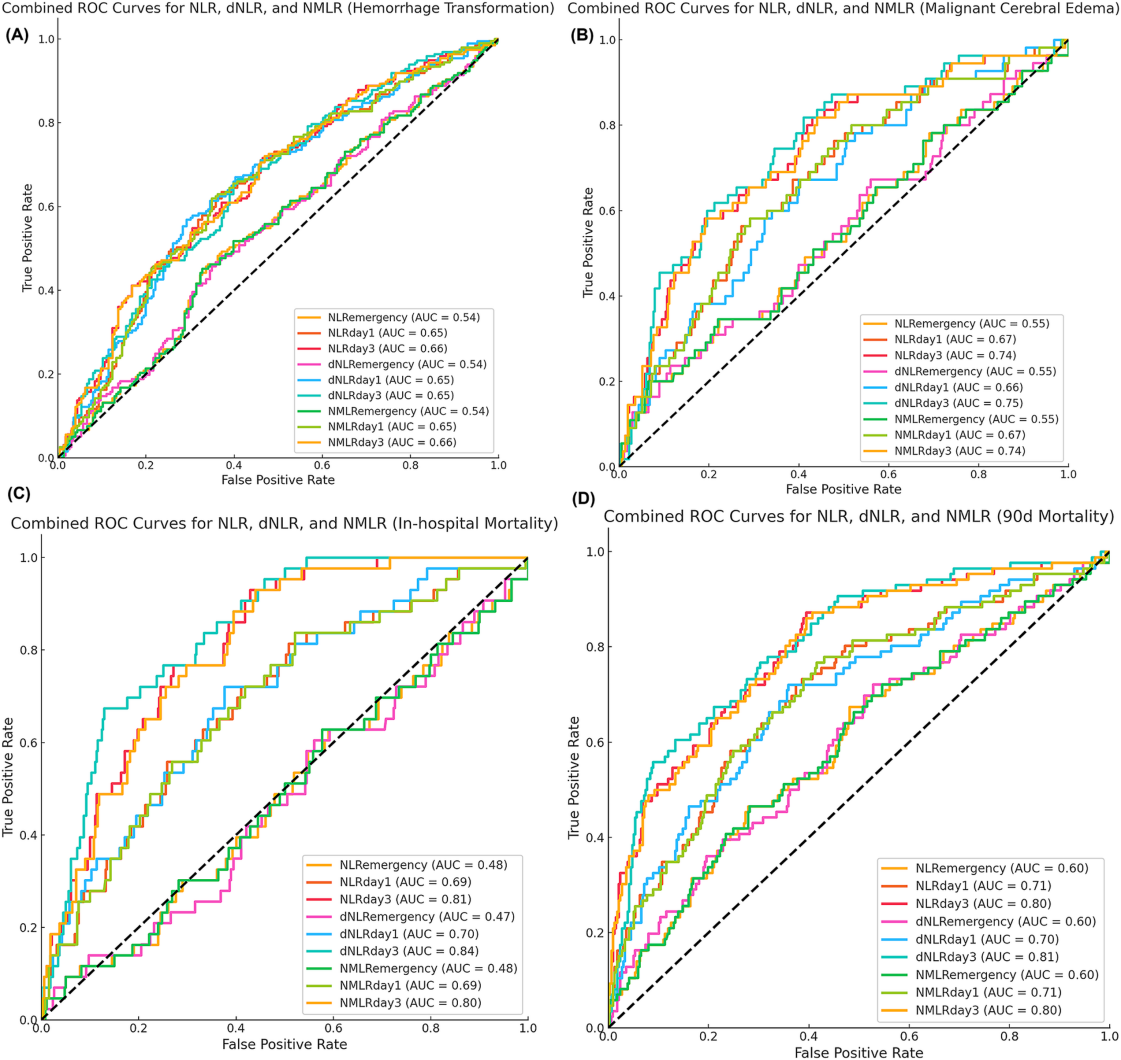
Predictive performance of inflammatory markers for secondary outcomes. Time-specific receiver-operating-characteristic curves for **(A)** hemorrhagic transformation, **(B)** malignant cerebral edema, **(C)** in-hospital mortality, and **(D)** 90-day all-cause mortality. Values in parentheses indicate area under the curve.

Receiver operating characteristic curve analysis (Table 3 and Figures 2, 3) revealed that the inflammatory markers on day 3 post-MT had the best predictive performance. The NLR on day 3 had an AUC of 0.85 (95% CI: 0.81–0.89) for the prediction of 90-day poor functional outcomes, which was significantly higher than that on day 1 post-MT [0.74 (0.70–0.79)] and on admission [0.65 (0.59–0.70)]. The optimal cut-off value for NLR on day 3 was 7.811, with corresponding accuracy, sensitivity, and specificity of 79, 87, and 75%, respectively. The dNLR and NMLR exhibited similar trends to NLR in predicting 90-day poor outcomes, with their predictive performance on day 3 [dNLR: AUC = 0.86; NMLR: AUC = 0.85] being significantly better than that on day 1 and day 0 (Figure 2).

**Table 3:** Comparison of laboratory parameters at different time points between good and poor outcome groups.

Outcome and parameter	AUC (95% CI)	Accuracy (95% CI)	Sensitivity (95% CI)	Specificity (95% CI)	Cut-off value
Poor outcome					
NLR day 0	0.65 (0.59–0.70)	0.60 (0.55–0.65)	0.73 (0.66–0.79)	0.52 (0.46–0.58)	4.428
NLR day 1	0.74 (0.70–0.79)	0.70 (0.65–0.74)	0.68 (0.61–0.75)	0.71 (0.65–0.76)	8.913
NLR day 3	0.85 (0.81–0.89)	0.79 (0.75–0.83)	0.87 (0.82–0.92)	0.75 (0.69–0.80)	7.811
dNLR day 0	0.64 (0.59–0.70)	0.59 (0.54–0.64)	0.75 (0.68–0.82)	0.49 (0.42–0.55)	3.371
dNLR day 1	0.73 (0.68–0.78)	0.70 (0.65–0.74)	0.62 (0.55–0.69)	0.75 (0.69–0.80)	5.086
dNLR day 3	0.86 (0.82–0.89)	0.78 (0.74–0.82)	0.88 (0.83–0.93)	0.71 (0.66–0.77)	4.643
NMLR day 0	0.65 (0.59–0.70)	0.60 (0.55–0.64)	0.74 (0.67–0.80)	0.50 (0.44–0.56)	5.028
NMLR day 1	0.75 (0.70–0.79)	0.70 (0.66–0.75)	0.67 (0.60–0.74)	0.72 (0.67–0.78)	9.316
NMLR day 3	0.85 (0.81–0.89)	0.80 (0.76–0.83)	0.84 (0.78–0.89)	0.77 (0.72–0.82)	7.969
Hemorrhage transformation					
NLR day 0	0.54 (0.49–0.60)	0.57 (0.52–0.62)	0.66 (0.60–0.72)	0.46 (0.39–0.53)	4.779
NLR day 1	0.65 (0.59–0.70)	0.63 (0.58–0.68)	0.63 (0.57–0.69)	0.63 (0.57–0.70)	9.741
NLR day 3	0.66 (0.61–0.71)	0.62 (0.57–0.67)	0.54 (0.47–0.60)	0.72 (0.65–0.78)	6.740
dNLR day 0	0.54 (0.49–0.60)	0.56 (0.51–0.61)	0.63 (0.57–0.69)	0.48 (0.41–0.55)	3.245
dNLR day 1	0.65 (0.60–0.70)	0.64 (0.60–0.69)	0.71 (0.65–0.77)	0.57 (0.50–0.64)	6.579
dNLR day 3	0.65 (0.60–0.71)	0.63 (0.58–0.67)	0.59 (0.53–0.66)	0.66 (0.60–0.73)	4.314
NMLR day 0	0.54 (0.49–0.60)	0.57 (0.52–0.62)	0.67 (0.61–0.73)	0.45 (0.38–0.52)	5.173
NMLR day 1	0.65 (0.59–0.70)	0.64 (0.59–0.68)	0.65 (0.59–0.71)	0.62 (0.55–0.69)	10.604
NMLR day 3	0.66 (0.61–0.71)	0.62 (0.57–0.67)	0.53 (0.47–0.60)	0.72 (0.66–0.78)	7.178
Malignant cerebral edema					
NLR day 0	0.55 (0.47–0.64)	0.84 (0.80–0.87)	0.93 (0.91–0.96)	0.20 (0.09–0.31)	14.317
NLR day 1	0.67 (0.60–0.75)	0.71 (0.66–0.75)	0.73 (0.68–0.77)	0.56 (0.43–0.69)	12.729
NLR day 3	0.74 (0.67–0.81)	0.78 (0.74–0.82)	0.81 (0.77–0.85)	0.58 (0.45–0.71)	13.53
dNLR day 0	0.55 (0.47–0.64)	0.80 (0.76–0.84)	0.89 (0.85–0.92)	0.24 (0.12–0.35)	7.335
dNLR day 1	0.66 (0.59–0.74)	0.61 (0.56–0.66)	0.60 (0.55–0.65)	0.67 (0.55–0.80)	6.392
dNLR day 3	0.75 (0.68–0.82)	0.62 (0.57–0.67)	0.59 (0.54–0.64)	0.82 (0.72–0.92)	4.775
NMLR day 0	0.55 (0.46–0.64)	0.84 (0.80–0.87)	0.93 (0.91–0.96)	0.20 (0.09–0.31)	15.049
NMLR day 1	0.67 (0.60–0.75)	0.69 (0.65–0.74)	0.71 (0.66–0.76)	0.58 (0.45–0.71)	13.039
NMLR day 3	0.74 (0.66–0.81)	0.78 (0.74–0.82)	0.81 (0.77–0.85)	0.58 (0.45–0.71)	14.107
In-hospital mortality					
NLR day 0	0.52 (0.42–0.61)	0.70 (0.65–0.74)	0.76 (0.72–0.80)	0.16 (0.05–0.27)	7.062
NLR day 1	0.69 (0.60–0.77)	0.60 (0.55–0.65)	0.59 (0.54–0.64)	0.72 (0.59–0.85)	10.561
NLR day 3	0.81 (0.75–0.87)	0.61 (0.56–0.65)	0.57 (0.52–0.62)	0.93 (0.85–1.00)	8.032
dNLR day 0	0.53 (0.43–0.62)	0.59 (0.54–0.64)	0.63 (0.58–0.68)	0.26 (0.13–0.39)	3.625
dNLR day 1	0.70 (0.62–0.78)	0.63 (0.59–0.68)	0.62 (0.57–0.67)	0.72 (0.59–0.85)	6.639
dNLR day 3	0.84 (0.79–0.89)	0.85 (0.81–0.88)	0.87 (0.84–0.90)	0.67 (0.53–0.81)	7.415
NMLR day 0	0.52 (0.42–0.61)	0.60 (0.55–0.65)	0.64 (0.59–0.69)	0.30 (0.17–0.44)	5.541
NMLR day 1	0.69 (0.60–0.77)	0.52 (0.47–0.56)	0.48 (0.43–0.53)	0.84 (0.73–0.95)	9.483
NMLR day 3	0.80 (0.75–0.86)	0.60 (0.55–0.65)	0.56 (0.51–0.61)	0.93 (0.85–1.00)	8.499
90-day mortality					
NLR day 0	0.60 (0.53–0.67)	0.55 (0.50–0.60)	0.52 (0.47–0.57)	0.67 (0.58–0.77)	3.935
NLR day 1	0.71 (0.64–0.77)	0.65 (0.60–0.69)	0.63 (0.57–0.68)	0.72 (0.63–0.82)	10.490
NLR day 3	0.80 (0.75–0.85)	0.66 (0.61–0.70)	0.61 (0.55–0.66)	0.87 (0.80–0.94)	7.877
dNLR day 0	0.60 (0.53–0.67)	0.52 (0.47–0.57)	0.47 (0.42–0.53)	0.72 (0.63–0.82)	2.544
dNLR day 1	0.70 (0.64–0.77)	0.66 (0.61–0.70)	0.64 (0.59–0.69)	0.72 (0.63–0.82)	6.451
dNLR day 3	0.81 (0.76–0.86)	0.81 (0.77–0.85)	0.87 (0.83–0.90)	0.60 (0.50–0.71)	6.915
NMLR day 0	0.60 (0.53–0.67)	0.67 (0.62–0.71)	0.72 (0.67–0.77)	0.47 (0.36–0.57)	5.950
NMLR day 1	0.71 (0.64–0.77)	0.62 (0.57–0.67)	0.58 (0.53–0.64)	0.77 (0.68–0.86)	10.46
NMLR day 3	0.80 (0.74–0.85)	0.66 (0.61–0.70)	0.61 (0.55–0.66)	0.86 (0.79–0.93)	8.499

NLR, neutrophil-to-lymphocyte ratio; dNLR, derived neutrophil-to-lymphocyte ratio; NMLR, neutrophil-monocyte-to-lymphocyte ratio.

For predicting hemorrhagic transformation, the performance of the NLR, dNLR, and NMLR was relatively weak (Figure 3A). Among them, the predictive value on day 3 was the highest [NLR: AUC = 0.66; dNLR: AUC = 0.65; NMLR: AUC = 0.66]. In predicting malignant cerebral edema, NLR, dNLR, and NMLR on day 3 had comparable performance [NLR: AUC = 0.74; dNLR: AUC = 0.75; NMLR: AUC = 0.74] (Figure 3B). The NLR, dNLR, and NMLR had higher predictive values for in-hospital mortality and 90-day mortality [NLR: AUC = 0.81 vs. AUC = 0.80; dNLR: AUC = 0.84 vs. AUC = 0.81; NMLR: AUC = 0.80 vs. AUC = 0.80] (Figures 3C,D).

Moreover, we observed an increasing trend in the predictive ability of inflammatory markers over time. The markers on admission usually performed the worst, while the markers on day 3 post-MT performed the best in predicting most outcomes. This trend was evident for all three inflammatory markers (NLR, dNLR, and NMLR).

### 3.4 Associations between the NLR and its derived indices and 90-day poor outcomes

To further explore the associations between the NLR and its derived indices at different time points with patients’ 90-day functional outcomes, we constructed three stepwise adjusted multivariable logistic regression models (Table 4). In the unadjusted crude model (Model A), the NLR, dNLR, and NMLR at all time points were significantly associated with 90-day poor outcomes (all *p* < 0.001). For each unit increase in the NLR, dNLR, and NMLR, the risk of 90-day poor outcomes increased by 41, 116, and 38%, respectively [NLR d0: OR = 1.41, 95% CI: 1.31–1.52; dNLR d0: OR = 2.16, 95% CI: 1.84–2.53; NMLR d0: OR = 1.38, 95% CI: 1.29–1.48].

**Table 4:** Univariable and multiple multivariable logistic regression model.

Variables	Model A		Model B		Model C	
	OR (95%CI)	*P-*value	OR (95%CI)	*P-*value	OR (95%CI)	*P-*value
NLR day 0	1.12 (1.06–1.18)	**<0.001**	1.13 (1.07–1.20)	**<0.001**	1.12 (1.06–1.19)	**<0.001**
NLR day 1	1.17 (1.12–1.22)	**<0.001**	1.16 (1.11–1.22)	**<0.001**	1.15 (1.10–1.20)	**<0.001**
NLR day 3	1.41 (1.31–1.52)	**<0.001**	1.41 (1.30–1.52)	**<0.001**	1.38 (1.28–1.49)	**<0.001**
dNLR day 0	1.19 (1.10–1.30)	**<0.001**	1.22 (1.12–1.33)	**<0.001**	1.20 (1.09–1.31)	**<0.001**
dNLR day 1	1.36 (1.24–1.49)	**<0.001**	1.34 (1.23–1.47)	**<0.001**	1.30 (1.19–1.42)	**<0.001**
dNLR day 3	2.16 (1.84–2.53)	**<0.001**	2.14 (1.82–2.51)	**<0.001**	2.04 (1.73–2.40)	**<0.001**
NMLR day 0	1.12 (1.06–1.17)	**<0.001**	1.13 (1.07–1.19)	**<0.001**	1.12 (1.06–1.18)	**<0.001**
NMLR day 1	1.16 (1.11–1.21)	**<0.001**	1.16 (1.11–1.21)	**<0.001**	1.14 (1.09–1.19)	**<0.001**
NMLR day 3	1.38 (1.29–1.48)	**<0.001**	1.38 (1.28–1.48)	**<0.001**	1.35 (1.26–1.45)	**<0.001**

Bold *P*-values indicate statistical significance (*P* < 0.05). OR, odds ratio; CI, confidence interval. Model A: unadjusted. Model B: adjusted for age, hyperlipidemia, and atrial Fibrillation. Model C: adjusted for variables in Model 2 plus number of baseline NIHSS, baseline GCS, number of thrombectomy attempts.

After further adjusting for age, hyperlipidemia, and atrial fibrillation (Model B), these associations persisted, and the effect sizes remained essentially unchanged. In the final model (Model C), we further adjusted for baseline NIHSS score, GCS score, and the number of thrombectomy attempts. The results indicated that all inflammatory markers still maintained significant associations with 90-day poor outcomes (both *p* < 0.001). The NLR, dNLR, and NMLR all retained strong independent predictive power, with each unit increase associated with a 38% (OR = 1.38, 95% CI: 1.28–1.49), 104% (OR = 2.04, 95% CI: 1.73–2.40), and 35% (OR = 1.35, 95% CI: 1.26–1.45) increased risk of poor outcomes, respectively. Furthermore, for all three inflammatory markers (NLR, dNLR, and NMLR), their ability to predict 90-day poor outcomes showed an increasing trend over time.

## 4 Discussion

This study systematically evaluated the dynamic changes in the NLR, dNLR, and NMLR and their prognostic predictive value in AIS patients after MT. The findings revealed that: (1) the NLR, dNLR, and NMLR in both groups peaked on day 1 post-MT and gradually declined, but the poor outcome group maintained higher levels, while the good outcome group showed a significant decreasing trend. (2) The NLR, dNLR, and NMLR on day 3 post-MT had stronger predictive power for 90-day prognosis compared to those on admission and day 1 post-MT. (3) Regarding secondary outcomes, the NLR, NMLR, and dNLR on day 3 post-MT had good predictive value for in-hospital mortality and 90-day all-cause mortality, but their predictive performance for hemorrhagic transformation and malignant cerebral edema was relatively weak. (4) After comprehensive adjustment for confounding factors, there was still a significant independent association between these inflammatory markers and 90-day poor outcomes.

Our study results are consistent with previous research findings. In a study of 204 AIS patients, Qian et al. (8) reported a similar dynamic change pattern in NLR: it gradually increased after onset, peaked at 24 h, and began to decline at 72 h. They also confirmed that the 72-h NLR was the optimal time point for predicting poor prognosis. Chen et al. (9) demonstrated that the 24-h NLR had stronger predictive value for 90-day functional outcomes and mortality than did baseline levels, but unfortunately, their study did not extend to the 72-h time point. The dynamic changes pattern in inflammatory markers may reflect the migration patterns of neutrophils after cerebral ischemia. Previous studies have shown that AIS can rapidly trigger a strong inflammatory cascade reaction. Ischemic brain tissue releases large amounts of chemokines and cytokines within hours of injury, leading to the recruitment and migration of peripheral circulating neutrophils to the ischemic region (15–17). The number of circulating neutrophils begins to increase 6 h after onset, reaches a peak within 24–48 h, and then gradually decreases after 72 h (18, 19). This time course is highly consistent with the dynamic changes in NLR observed in our study.

Notably, the poor outcome group maintained higher inflammatory levels on day 3 post-MT, while the good outcome group showed a significant decreasing trend. These finding suggest that patients with poor prognoses may have persistent inflammatory responses and immune imbalance. As a marker of systemic inflammatory response, an elevated NLR reflects the dual effects of innate immune activation (neutrophil increase) and adaptive immune suppression (lymphocyte decrease). Previous studies have shown that neutrophils can participate in the pathological process of ischemic brain injury through multiple mechanisms (20, 21). First, neutrophils are the main source of matrix metalloproteinase-9 (MMP-9). MMP-9 can directly act on the tight junction proteins between vascular endothelial cells, thereby opening the BBB on the luminal side of blood vessels or acting on the vascular basement membrane through endocytosis, thus exacerbating the neuroinflammatory response (22). Second, neutrophils release various cytotoxic factors, such as reactive oxygen species, myeloperoxidase, proteases, and inflammatory mediators (23). The combined action of these factors leads to damage to the neurovascular unit and expansion of brain tissue injury. In addition, neutrophils also promote microthrombus formation through interactions with platelets and coagulation factors, and may interfere with the recovery of local cerebral blood flow by adhering to the microvasculature (24).

Conversely, lymphocyte count changes reflect the body’s stress state and immune function. Lymphocytopenia suggests the occurrence of glucocorticoid-mediated stress responses and sympathetic nervous system activation, which may exacerbate ischemic injury (25). Different lymphocyte subsets play complex regulatory roles in the progression of ischemic stroke: γδ T cells and CD8+ T cells aggravate tissue damage by releasing pro-inflammatory factors, while regulatory T cells exert neuroprotective effects by suppressing inflammatory responses and maintaining immune homeostasis (26–28).

As a novel inflammatory marker, NMLR provides a more comprehensive assessment of the inflammatory state by integrating the monocyte count (29). Studies have shown that AIS patients commonly exhibit elevated neutrophil and monocyte counts accompanied by decreased lymphocyte counts. Among them, monocytes can activate platelets to form platelet–monocyte aggregates, promoting the release of inflammatory and vasoactive substances and affecting hemodynamics, thereby exacerbating ischemic brain injury (23). This viewpoint is supported by Dragu et al.’s (30) study, which revealed that an elevated baseline monocyte count was associated with mortality risk in patients with acute myocardial infarction. Although theoretically integrating monocyte indicators may improve predictive ability, in our study, the predictive performance of the NMLR was comparable to that of the NLR and dNLR. Additionally, Liu et al. (26) confirmed that the dNLR could independently predict prognosis in patients with coronary heart disease after percutaneous coronary intervention, and our study further validated the application value of the dNLR in prognostic assessment after MT. However, we noted that there were no significant differences in predictive value among the NLR, dNLR, and NMLR.

On the basis of the key role of inflammatory responses in stroke prognosis revealed in our study, therapeutic interventions targeting immune-inflammatory reactions may provide new directions for improving patient outcomes. Although related research has made some progress, clinical translation still faces challenges. Currently, studies on the ability of fingolimod combined with alteplase to improve neurological deficits by regulating circulating lymphocyte levels have shown potential value but still require validation in large-scale randomized controlled trials (31). In basic research, dextromethorphan has exhibited significant anti-inflammatory effects by inhibiting the expression of TNF-*α*, iNOS, IL-1β, and COX-2, whereas edaravone alleviates secondary neurological injury by scavenging oxygen free radicals (32, 33). These research advances suggest that multitarget intervention strategies targeting inflammatory responses may open new avenues for the treatment of ischemic stroke, but their clinical application value still needs further exploration. Therefore, in combination with our study results, for patients with persistently high inflammatory markers on day 3, treatment regimens targeting immune–inflammatory responses may help improve patient prognosis, but this hypothesis still needs to be confirmed through large-scale, prospective clinical studies.

This study has several limitations that need to be noted. First, as a single-center retrospective study, the generalizability of the results may be limited. In particular, the inclusion criteria requiring complete day 3 laboratory examination data may lead to selection bias, as some patients with early death or extremely severe clinical conditions may be excluded from the analysis. Second, although we identified the optimal predictive thresholds for inflammatory markers, the external validation and clinical application value of these cutoff values remain to be confirmed. Moreover, this study was unable to provide specific recommendations for individualized treatment strategies for patients with different inflammatory levels.

## 5 Conclusion

This study confirmed that inflammatory markers (NLR, dNLR, and NMLR) on day 3 post-MT can serve as independent predictors of prognosis in AIS patients treated with MT, especially those with strong predictive value for poor 90-day functional outcomes, in-hospital mortality, and 90-day mortality. Notably, patients with a poor prognosis maintained increased inflammatory levels on day 3 post-MT, whereas patients with a good prognosis presented a significant decreasing trend. This dynamic change pattern may provide a new perspective for clinical risk assessment. These findings emphasize the importance of dynamic monitoring of inflammatory markers after MT and may offer new strategic evidence for early risk stratification and individualized treatment plan formulation in AIS patients.

## Data Availability

The raw data supporting the conclusions of this article will be made available by the authors, without undue reservation.
